# Sildenafil Supplementation for Women Undergoing Infertility Treatments: A Systematic Review and Meta-Analysis of Randomized Controlled Trials

**DOI:** 10.3390/jcm10194346

**Published:** 2021-09-24

**Authors:** Loris Marin, Alessandra Andrisani, Luciana Bordin, Francesco Dessole, Marco Noventa, Amerigo Vitagliano, Giampiero Capobianco, Guido Ambrosini

**Affiliations:** 1 Department of Women’s and Children’s Health, University of Padua, 35128 Padua, Italy; loris.marin@unipd.it (L.M.); alessandra.andrisani@unipd.it (A.A.); vitagliano@gmail.com (A.V.); guido.ambrosini@unipd.it (G.A.); 2 Department of Molecular Medicine-Biological Chemistry, University of Padova, 35131 Padova, Italy; luciana.bordin@unipd.it; 3 Department of Surgical, Microsurgical and Medical Sciences, Gynecologic and Obstetric Clinic, University of Sassari, 07100 Sassari, Italy; francescodessole@gmail.com (F.D.); capobianco@gmail.com (G.C.)

**Keywords:** Sildenafil, endometrial thickness, timed intercourse, intrauterine insemination, in vitro fertilization

## Abstract

The aim of this systematic review and meta-analysis is to summarize data on the effectiveness of Sildenafil supplementation for women undergoing assisted reproduction techniques. This meta-analysis of randomized controlled trials (RCTs) evaluates the effects of Sildenafil administration during infertility treatments compared with a control group in infertile women. Outcomes evaluated were endometrial thickness (ETh) and the clinical pregnancy rate (CPR). The chemical pregnancy rate (ChPR) was also evaluated. Pooled results were expressed as the risk ratio (RR) or mean differences (MD) with a 95% confidence interval (95% CI). Women undergoing ovulation induction who received Sildenafil showed higher ETh and a higher CPR in comparison to controls. In this group, both the ETh and ChPR resulted in significantly higher values only with delayed start administration. Women undergoing fresh or frozen embryo transfer who received Sildenafil showed no significant advantages regarding ETh and CPR in comparison to controls. In this group, we found a significantly higher ChPR in women receiving Sildenafil. A subgroup analysis revealed significant advantages regarding ETh with oral administration for women undergoing fresh or frozen embryo transfer. Sildenafil therapy appears to improve endometrial thickness and pregnancy rate in women undergoing timed intercourses but it resulted not effective in IUI and IVF treatments. Further RCTs with rigorous methodology are still mandatory.

## 1. Introduction

Despite advances in assisted reproductive technologies (ARTs), the cumulative success rate of the procedures remains suboptimal, with an estimated overall pregnancy rate of around 30% [1]. According to several studies, one of the major limiting factors for the success of ART might be represented by impaired endometrial receptivity (ER) [2].

Despite all efforts to validate specific and effective markers to find an optimal window of implantation for embryo transfer [3], endometrial thickness (ETh) is still considered the best surrogate measurement and a crucial factor for implantation. Accordingly, several studies reported a direct correlation between low ETh (<7 mm) and low success rates of ARTs—and medically assisted reproduction—(MAR) procedures, including intrauterine insemination (IUI) and in vitro fertilization (IVF) with fresh embryo transfer (fresh-ET) or frozen embryo transfer (frozen-ET) [4,5,6].

During the last decades, several strategies (including hormonal and non-hormonal adjuvants) have been tested with the purpose of increasing ETh in women undergoing infertility treatments, with conflicting results [7,8,9,10,11,12,13,14,15,16,17].

One of the adjuvants that showed potential beneficial effects on endometrial thickening in women undergoing infertility treatments is sildenafil citrate, a 5-phosphodiesterase inhibitor widely used for male erectile dysfunction [18]. The Sildenafil pharmacological effect of Sildenafil is based on the prevention of cGMP breakdown thereby increasing smooth muscle relaxation and vasodilation [18]. At the endometrium level, Sildenafil may increase uterine artery flow and exert a positive effect on endometrial growth in response to estrogenic stimulation [19]. Moreover, it may improve endometrial tolerance to the embryo through decreasing local natural killer cell activity and favoring the accomplishment of proper embryo implantation [20]. Mechanisms of action of Sildenafil on the endometrium are not fully understood. Due to its supposed action of favoring implantation both through the increase in endometrial thickness [19] and through the immune action [20], this type of add-therapy has been studied on different types of infertile women, both those with a thin endometrium and those without an apparent endometrial problem. In fact, implantation does not occur in about 1/3 of transfers of euploid blastocyst in women without an apparent endometrial abnormality [21].

Based on these principles, randomized controlled trials investigated the efficacy of oral or vaginal Sildenafil administration in women undergoing infertility treatments. Thus, the aim of this present systematic review and meta-analysis was to summarize the current evidence on the effectiveness of Sildenafil administration for improving ETh and the success of ARTs and MAR procedures.

## 2. Material and Methods

### 2.1. Study Design

This is a systematic review and meta-analysis of RCTs evaluating the effectiveness of add-therapy with Sildenafil in MAR and ARTs procedures. The study protocol was registered in PROSPERO before the start of the literature search (CRD42020165583). The review was written following the Preferred Reporting Items for Systematic Reviews and Meta-Analyses (PRISMA) guidelines [22].

### 2.2. Search Strategy

A literature search was conducted on electronic databases (Medline, Scopus, Embase, ScienceDirect, the Cochrane Library, Clinicaltrials.gov, Cochrane Central Register of Controlled Trials, EU Clinical Trials Register and World Health Organization International Clinical Trials Registry Platform) up to April 2021. Key search terms included the following: “sildenafil” (Mesh) AND “infertility” OR “timed intercourse” OR “intrauterine insemination” OR “in-vitro fertilization” OR “assisted reproductive technology” OR “controlled ovarian stimulation” OR “fresh embryo transfer” OR “frozen embryo transfer”.

### 2.3. Inclusion Criteria

- Language: studies reported in English language
- Study designs: randomized controlled trials
- Population: infertile women undergoing MAR and ARTs procedures, including TI, IUI and IVF with fresh fresh-ET or Frozen-ET.
- Intervention: Sildenafil therapy
- Timing of intervention: during the monitored cycle for TI or IUI; during the course of controlled ovarian stimulation (COS) for IVF and fresh_ET and during the course of endometrial preparation for frozen_ET.
- Comparator: infertile women with unexplained infertility and with ovulatory or anovulatory cycles, with thin endometrium or with multiple failed ART cycles, undergoing TI or IUI or COS with fresh_ET embryo transfer or endometrial preparation for frozen-ET
- Outcomes and their definitions: *endometrial thickness* (ETh-transvaginal ultrasonography measurement of the endometrium at the maximal distance between each myometrial/endometrial interface in a prearranged moment of the menstrual cycle), *clinical pregnancy rate* (CPR-per woman randomized, defined as the presence of a gestational sac on transvaginal ultrasound), *chemical pregnancy rate* (ChPR-per woman randomized, defined as serum measurement of beta Human chorionic gonadotropin >5 mU/mL).

### 2.4. Study Selection and Data Extraction

Two authors (L.M., A.V.) independently screened titles and abstracts of studies obtained by the search strategy. The text of each potentially relevant study was assessed for inclusion in the review, independently by the two authors. A manual search of reference lists of retrieved studies and available review articles was also performed to avoid missing relevant publications. The same authors have independently extracted data from the studies. One other author (A.A.) reviewed the selection and data extraction process. We searched for published and unpublished studies from the aforementioned electronic databases. The results were compared, and any disagreement was resolved by consensus.

### 2.5. Risk of Bias

Two authors (L.M., A.V.) independently judged the methodological quality of the included studies by using the criteria reported in the Cochrane’s Handbook for Systematic Reviews of Interventions. Seven specific domains related to risk of bias were assessed, which are as follows: random sequence generation, allocation concealment, blinding of participants and personnel, blinding of outcome assessment, incomplete outcome data, selective data reporting and other bias.

Authors’ judgements were expressed as “low risk”, “high risk”, or “unclear risk” of bias. For the estimation of “selective data reporting”, we evaluated study protocols, when available. If protocol was not available, studies have been judged at high risk of bias.

### 2.6. Statistical Analysis

Two authors (M.N, L.M.) independently performed the data analysis using Review Manager (version 5.3). All analyses were performed with the random-effects model (by DerSimonian and Laird), with an intention-to-treat approach. Variables were compared using the risk ratio (RR) or mean differences (MD), with a 95% confidence interval (95% CI). A *p* value lower than 0.05 was considered as statistically significant. Heterogeneity was measured with the Higgins I2.

When possible, we performed a subgroup analysis to evaluate the influence of intervention (Sildenafil treatment) based on the timing of administration (started with ovarian stimulation versus delayed start, 7–8 days from ovarian stimulation) or the route of administration (vaginal versus oral route).

Concerning specific outcomes, in cases of studies with missing data about ChPR but data about CPR, we decided to consider CPR as a surrogate measurement of ChPR.

Risk of bias across studies was not measured due to the low number of studies included (according to the Cochrane’s Handbook recommendation).

## 3. Results

### 3.1. Study Selection

The electronic searches provided a total of 656 citations. After the removal of 100 duplicate records, 556 citations remained. Of these, 528 records were excluded after title/abstract screening (not relevant to the review). We examined the full text of 28 manuscripts, and, of these, we excluded 16 papers—two trials investigated Tadalafil as the 5-phosphodiesterase inhibitor [23,24], two trials reporting not analyzable data (only abstracts available) [25,26], four papers because the design was retrospective [27,28,29,30], six because of the observational design [31,32,33,34], one paper because it was a review [35]. Another paper was excluded because the results of Sildenafil efficacy in women with unexplained infertility were provided based on a subgroup analysis (duration of infertility) only, and the size of the subgroups was not described [36].

**Figure 1:**
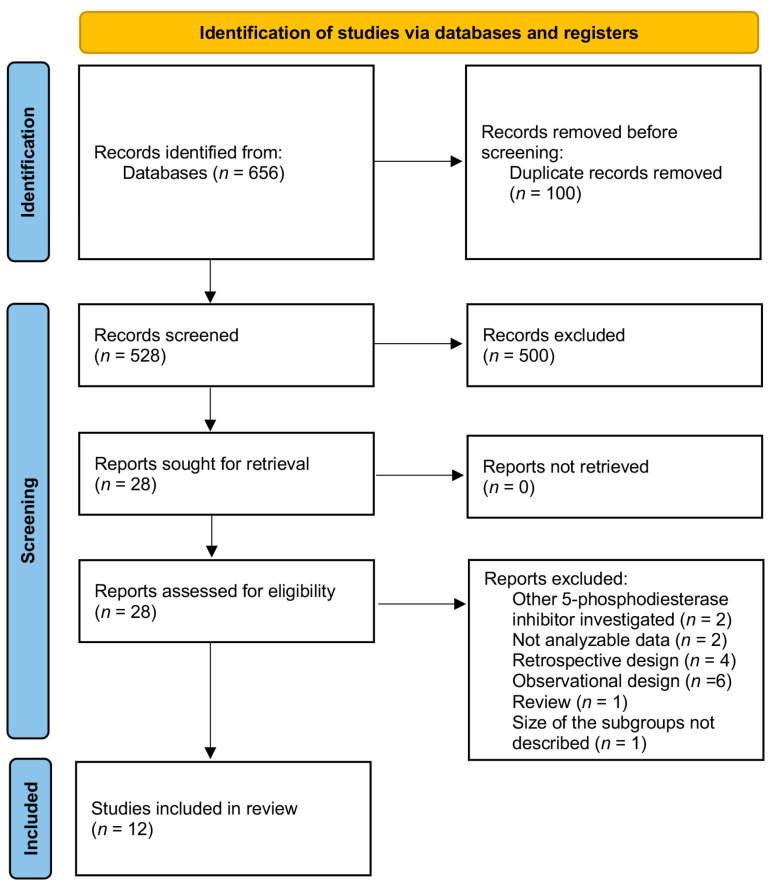
Prisma Flow Diagram.

Finally, 12 manuscripts were included in the meta-analysis (see Figure 1: Prisma Flow Diagram). We analyzed all outcomes according to the assisted reproductive technique used. Considering the number of included studies, we performed analyses firstly together and then separately by TI/IUI (TI/IUI group) [19,37,38,39,40,41] and IVF fresh-ET and frozen-ET (IVF fresh-ET/frozen-ET group) [12,42,43,44,45,46]. Full details about the included studies are available in Table 1 and Table 2.

### 3.2. TI/IUI Section

#### 3.2.1. Included Studies

Six RCTs have been included in the TI/IUI section with a total of 460 participants (Table 1). Four studies [37,38,40,41] did not report random sequence generation; one study [39] used simple randomization; one study [19] used computer number generation by the closed envelope method for randomization. All those studied did not report blindness of participants and personnel. Two studies were placebo-controlled [19,37]; in one study, the control group received co-intervention with estradiol [38], and the remaining were treatment versus no treatment [39,40,41]. All six studies assessed endometrial thickness by ultrasound on day 13 or 14 of the cycle or on the hCG trigger day. Three trials [38,39,41] assessed CPR, and the other three trials [19,37,40] evaluated ChPR. Two studies [38,40] assessed the pregnancy rate on cumulative cycles (three).

#### 3.2.2. Patients

Inclusion criteria were different among studies. Three studies enrolled women with unexplained fertility, with ovulatory cycles [19,37,40]. One study considered women affected by polycystic ovarian syndrome [39]. Two studies only considered infertile women with a persistently thin endometrium, defined as endometrium thickness <7mm on the 8th day of the cycle or on the day of ovulation trigger [38,41]. Four studies used clomiphene citrate at different dosages (50–150 mg) for 5 days during the menstrual cycle [19,37,40,41]. Mangal S. et Mahirishi S. [38] used both clomiphene citrate and gonadotropins for follicle recruitment and instead Mohamed T.Y. used letrozole [39]. In all studies, ovulation induction was performed with human chorionic gonadotropin. Only one study [19] described the luteal phase support with oral progesterone from the day of ovulation (Dufaston 10 mg twice daily). When considering outcomes, in two studies [38,40] authors calculated the cumulative pregnancy rate in three cycles.

#### 3.2.3. Type, Dose and Duration of Intervention

In all except one trial [38], Sildenafil was orally administered. The daily dose of Sildenafil varied among studies; 25 mg daily orally [41], 50 mg daily orally [39,40] or 75 mg daily orally [19,37] and 100 mg/day vaginally [38]. The timing of administration was from the 7th and 8th days until ovulation induction in four trials [37,38,39,40], and just in two studies, the administration started from the beginning of the menstrual cycle (1st–2nd day) [19,41].

### 3.3. IVF Fresh-ET/Frozen-ET Section

#### 3.3.1. Included Studies

Six RCTs have been included in the IVF Fresh-ET/Frozen-ET section (Table 2) with a total of 393 participants. Three RCTs reported data about COS with fresh embryo transfer with 198 patients included [42,45,46], and three RCTs reported data about FET with 195 patients included [12,43,44].

One study [46] did not report random sequence generation, and in three studies [12,43,45], an adequate method of random sequence generation was used, and the randomizing methods of last two studies [42,44] were judged at high risk of bias. Two studies [42,45] were double blinded, and four studies [12,43,44,46] did not report the blindness of participants and personnel. Two studies were placebo-controlled [42,45]; three [12,44,46] were treatment versus no treatment, and the remaining study [43] was vaginal Sildenafil vs. vaginal estradiol.

All six studies assessed endometrial thickness on the hCG trigger day or before progesterone administration starting. One trial [43] outcome was only endometrial thickness. Regarding the pregnancy rate, one study [12] evaluated ChPR, whereas the other three [42,45,46] assessed CPR, and another one [44] considered both ChPR and CPR.

#### 3.3.2. Patients

Concerning COS and fresh ET, the inclusion criteria were different among trials. One study [46] enrolled infertile women with at least two previous failed FIVET cycles. Another study [45] considered women with at least two previous failed FIVET cycles and also a persistent thin endometrium defined as endometrial thickness < 7 mm measured the day of ovulation triggering. Moreover, in the latter study, enrolled women needed to have an adequate ovarian reserve defined as AMH > 1.5 ng/mL and FSH < 10 UI/mL. One trial [42] enrolled poor responders women with age ≤ 35 and no previous ovarian surgery.

Concerning studies analyzing FET, all included patients reported an antecedent of a poor endometrial response during the previous cycle for frozen-thawed embryo transfer [12,43,44]. In all three studies, artificial cycles were used for endometrial preparation. Estradiol was administered by a step-up method in two studies [12,43]; instead, in one study [44], oral estradiol was administered in a fixed dose (2 mg every 6–8 h) from day 2 of the menstrual cycle.

#### 3.3.3. COS Cycles

Considering only trials that evaluated fresh-ET, two studies [45,46] evaluated women undergoing a long GnRH-agonist protocol with a starting dose of gonadotropins defined according to women characteristics, while in one trial [42], a short-antagonist protocol with a high starting dose of gonadotropins was employed. In all trials [42,45] except one (in which data were not specified [46]), embryo transfer was performed after two–three days of an in vitro culture. Concerning luteal phase support (LPS), in one trial, a daily intramuscular injection of progesterone (150 mg) was administered [42]; instead, in another study, LPS was indistinctly supported through the daily intramuscular or vaginal administration of progesterone [45]. Finally, the remaining trial did not specify the type of LPS [46].

#### 3.3.4. Type, Dose and Duration of Intervention

Considering only trials that evaluated fresh-ET, in all except one trial (using oral Sildenafil, 50 mg [42]), Sildenafil was vaginally administered [45,46]. The daily dose of vaginal Sildenafil was 100 mg/day in both studies. The timing of administration was from the 1st–2nd day until an ovulation trigger or oocyte retrieval all trials [42,45,46]. In one of these three trials [45], one of the three randomized arms received a placebo from the from 1st–2nd day until 2 days before hCG administration, then Sildenafil until OPU.

Concerning studies analyzing FET, in all trials [12,43,44], Sildenafil was vaginally administered. The daily dose of Sildenafil varied among studies; two trials administered 100 mg/day [43,44], one trial 50 mg/day [12]. The timing of administration was from the beginning of the menstrual cycle (2nd–3rd day) until approximately the start of progesterone administration [12,43,44].

### 3.4. Risk of Bias

Random sequence generation: four studies [12,19,43,45] were judged at low risk of bias because an adequate method of random sequence generation was reported. Five studies were judged at unclear risk of bias (no information reported) [37,38,40,41,46], and three trials were judged at high risk of bias because simple randomization was used [39,42,44].

Allocation concealment: two studies were judged at low risk of bias [19,37]; one trial was judged at high risk of bias [42], while no information was reported in other studies [12,38,39,40,41,43,44,45,46].

Blinding of participants and personnel: two studies were at low risk of bias [42,45]; all other trials were at unclear risk because no information was reported [12,19,37,38,39,40,41,43,44,46].

Blinding of outcome assessment: in all studies, the blinding of the assessor was not specified. Because of the objective outcome, assessor knowledge could hardly interfere with the results, and studies were judged at low risk [12,19,37,38,39,40,41,42,43,44,45,46].

Incomplete outcome data: all studies were judged at high risk of bias because none of them reported data about the live birth rate and ongoing pregnancy rate [12,19,37,38,39,40,41,42,43,44,45,46].

Selective data reporting: five studies were judged at high risk of selective data reporting due to the absence of the registered study protocol [37,38,39,40,41,42,43,44,46]. The remaining studies were judged at low risk [12,19]. For one study [45], there was inconsistency between the planned study start (recorded protocol) and the effective study start.

Other bias: in all studies, a power analysis was missing [12,19,37,38,39,40,41,42,43,44,45,46], and in one study [45], there was inconsistency between the population characteristic and inclusion criteria.

### 3.5. Adverse Effects

No trial reported adverse effects resulting from the intervention.

### 3.6. Effects of Intervention (TI/IUI Section)

**Figure 2:**
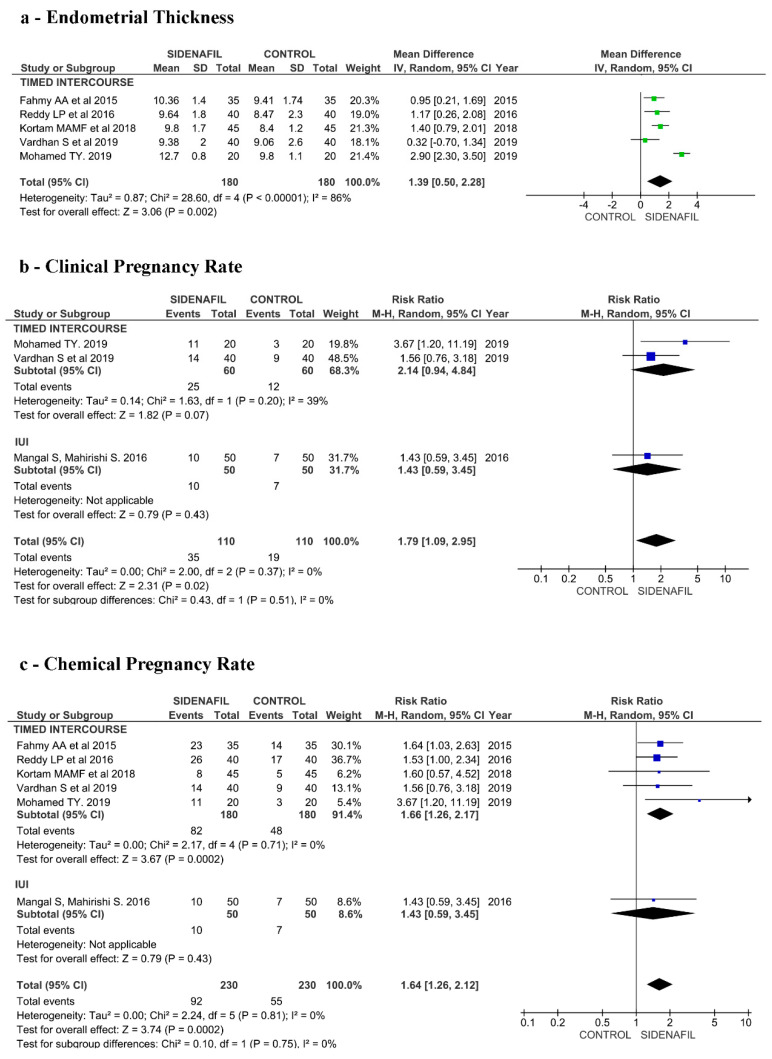
(**a**) Forest plot of comparison: endometrial thickness (ETh) according to group allocation—intervention (Sildenafil) versus control in intrauterine insemination and timed intercourse cycles. (**b**) Forest plot of comparison: clinical pregnancy rate (CPR) according to group allocation—intervention (Sildenafil) versus control in intrauterine insemination and timed intercourse cycles. (**c**) Forest plot of comparison: chemical pregnancy rate (ChPR) according to group allocation—intervention (Sildenafil) versus control in intrauterine insemination and timed intercourse cycles.

Concerning ETh, the analysis included a total number of 360 participants from altogether five studies [19,37,39,40,41], all analyzing TI. The only study analyzing IUI did not report this outcome [38]. Women receiving Sildenafil therapy showed higher ETh in comparison to controls, MD 1.39 (95% CI 0.50–2.28, *p* = 0.002), but with high statistical heterogeneity (I2 = 86%). See Figure 2a.

Concerning CPR, three papers reported this outcome [38,39,41], one analyzing IUI [38] and two TI [39,41]. We found a significant advantage related to Sildenafil treatment with a total RR of 1.79 (95% CI 1.09–2.95, *p* = 0.02) with no heterogeneity (I2 = 0%). The separate analysis of TI and IUI showed no statistical significance. See Figure 2b.

Concerning ChPR, six papers reported this outcome [19,37,38,39,40,41], one analyzing IUI [38] and five TI [19,37,39,40,41]. We found a significant advantage related to Sildenafil treatment with a total RR of 1.64 (95% CI 1.26–2.12, *p* = 0.0002), with no statistical heterogeneity (I2 = 0%). Considering only TI, the RR was 1.66 (95% CI 1.26–2.17, *p* = 0.0002), but no significant advantages were found in the paper reporting data from IUI cycles. See Figure 2c.

Subgroups analysis:

Concerning administration route (vaginal vs. oral), five papers [19,37,39,40,41] reported data from oral administration and only one paper from vaginal [38]. As the only study evaluating vaginal administration in this subgroup (TI/IUI) [38] compared Sildenafil to estrogen, while the other subgroup studies compared Sildenafil to no intervention, we excluded this study for this analysis.

Concerning the timing of Sildenafil administration (at the beginning of stimulation versus delayed start) and ETh, three papers reported data about delayed start [37,39,40] and two papers about the beginning of stimulation [19,41]. Significant beneficial effects in terms of ETh seemed related to delayed start (MD 1.70, 95% CI 0.36–3.03, I2 = 90%, *p* = 0.01); on the contrary, administering Sildenafil at the beginning of the stimulation seems not effective (MD 0.94, 95% CI -0.11–1.99, I2 = 69%, *p* = 0.08). See Supplemental Figure S1a.

Concerning the timing of Sildenafil administration (at the beginning of stimulation versus delayed start) and ChPR, four papers reported data about delayed start [37,38,39,40] and two papers about the beginning of stimulation [19,41]. ChPR results were significantly higher with delayed start [37,38,39,40] (RR 1.65, 95% CI 1.24–2.20, I2 = 0%, *p* = 0.0006). On the contrary, there was not a significant ChPR increase when Sildenafil administration starts at the beginning of stimulation [19,41] (RR 1.57, 95% CI 0.87–2.83 I2 = 0%, *p* = 0.13). See Supplemental Figure S1b.

### 3.7. Effects of Intervention (IVF Fresh-ET/Frozen-ET Section)

**Figure 3:**
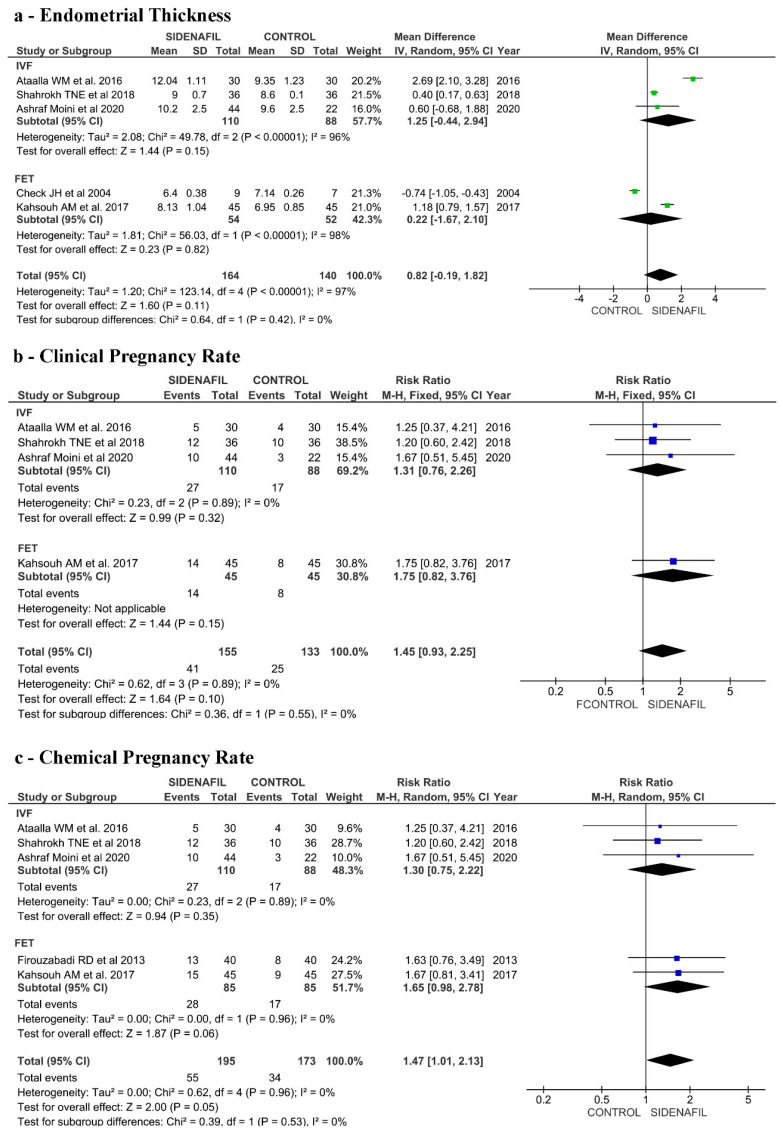
(**a**) Forest plot of comparison: endometrial thickness (ETh) according to group allocation—intervention (Sildenafil) versus control in IVF fresh and frozen embryo transfer. (**b**) Forest plot of comparison: clinical pregnancy rate (CPR) according to group allocation—intervention (Sildenafil) versus control in IVF fresh and frozen embryo transfer. (**c**) Forest plot of comparison: chemical pregnancy rate (ChPR) according to group allocation—intervention (Sildenafil) versus control in IVF fresh and frozen embryo transfer.

Concerning ETh, five papers reported this outcome [42,43,44,45,46], three of them reported data from IVF fresh-ET (IVF group) [42,45,46] and two from frozen-ET (FET group) [43,44], with a total of 313 participants. Considering together fresh- and frozen-ET, we did not find a significant advantage in the intervention group in comparison to controls (MD 0.82, 95% CI −0.19–1.82, *p* = 0.11). When we evaluated separately the two groups, a significant advantage in the intervention group in comparison to controls was absent both in the IVF group (MD 1.25, 95% CI −0.44–2.94, *p* = 0.15) and in the FET group (MD 0.22, 95% CI −1.67–2.10, *p* = 0.82). See Figure 3a.

Concerning CPR, four papers reported this outcome [42,44,45,46], three of them reported data from IVF fresh-ET (IVF group) [42,45,46] and only one from frozen-ET (FET group) [44], with a total of 293 participants. We found no significant increase in the CPR in the intervention group compared to controls analyzing together (RR 1.45, 95% CI 0.93–2.25) and separately by IVF and FET group. In particular, a significant advantage in the intervention group in comparison to controls was absent in the IVF group (RR 1.31, 95% CI 0.76–2.26) and in the FET group (RR 1.75, 95% CI 0.82–3.76). See Figure 3b.

Concerning ChPR, analyzing together IVF fresh-ET and frozen-ET, we found a significantly higher ChPR in the intervention group compared to controls (RR 1.47, 95% CI 1.0–2.13, *p* = 0.05), with no heterogeneity (I2 = 0%). A separate analysis was not significant. We found no significant increase in the ChPR neither in the intervention group of women who underwent IVF cycles compared to controls (RR 1.30, 95% CI 0.75–2.22, *p* = 0.89) nor in the intervention group of women who underwent FET cycles compared to controls (RR 1.65, 95% CI 0.98–2.78, *p* = 0.96). See Figure 3c.

Subgroup analysis:

Concerning administration route (vaginal vs. oral) and ETh, we found no significant effect of Sildenafil on endometrial thickness (MD 0.33, 95% CI −0.54–1.20, *p* = 0.45), but heterogeneity was high (I2 = 95%). See Supplemental Figure S2a.

Concerning administration route (vaginal vs. oral) and ChPR, a significant increase in the ChPR was found from vaginal administration, with a RR of 1.49, (95% CI 1.00–2.21, *p* = 0.05). No significant benefits in terms of ChPR seem to be associated to oral administration, even if only one study was included in this section [13]. See Supplemental Figure S2b.

## 4. Discussion

### 4.1. General Considerations

Despite the improvement in ARTs, the live birth rate is still low even if top-quality embryos are obtained [47]. A great issue is the obtaining of a receptive endometrium [48,49]. Despite new technologies that allow the detection of the implantation window through an endometrial biopsy performed during the previous menstrual cycle [50,51], the widely used method in the clinical practice to establish if an endometrium is suitable for implantation is the transvaginal ultrasound evaluation of ETh [52,53,54].

The underlying cause of the thin endometrium must be sought and resolved before attempting another cycle for achieving pregnancy. Hysteroscopy is usually the gold standard as a second-line diagnostic investigation [55,56,57], and Asherman’s syndrome is the first pathological condition that must be excluded [58]. Another underlying underdiagnosed condition that has recently been re-examined might be chronic endometritis that can be suspected with diagnostic hysteroscopy and confirmed with endometrial biopsy [59,60,61,62].

Despite these second-line investigations, many times the underlying cause cannot be identified. Although sometimes the endometrium might be receptive even if a thinner value is used as a cut-off [49], an association has been demonstrated between low endometrial thickness, ART failure and adverse pregnancy outcomes related to an abnormal placentation such as hypertension, preeclampsia, intrauterine growth defects [63,64,65].

For these reasons, many efforts have been made in order to obtain a thicker endometrium with different strategies involving hormonal approaches (estradiol administration adjustment, hCG administration during the follicular phase, GnRH agonist administration during the luteal phase), the intrauterine infusion of growth factors such as the granulocyte colony-stimulating factor and platelet-rich plasma and the usage of factors that act on endometrium vascularity [66]. For this latter approach, low dose aspirin has been used and also phosphodiesterase inhibitors [66]. Among phosphodiesterase inhibitors, there are non-specific inhibitors such as pentoxifylline and selective ones such as phosphodiesterase type 5 inhibitor (Tadalafil and Sildenafil) [24,67]. However, despite different approaches that might be available, a lack of solid evidence in the published literature limits their clinical applicability.

In particular, the usage of selective phosphodiesterase type 5 inhibitors seems a promising strategy supported by a valid biological rationale. Indeed, Sildenafil causes vasodilatation preventing cGMP breakdown and increasing relaxation of the smooth muscle [12,45]. This effect is widely used in males for erectile dysfunction [68]. With a similar mechanism, Sildenafil might increase uterine artery flow with subsequent enhanced endometrial vascularization and improved endometrial growth under estrogenic influence [19,45].

### 4.2. Main Findings

In this meta-analysis, we tested the effect of Sildenafil as an add-therapy during the TI or IUI cycle and during IVF and fresh-ET or frozen-ET on ETh and the pregnancy rate. We found that Sildenafil supplementation significantly improves ETh when administered during a timed intercourse or IUI cycles; on the contrary, it does not seem to take a significant advantage when administered during fresh-ET or frozen-ET. In both groups, there was a bad consistency (I2 86% and 97%, respectively). Similarly, analyzing TI or IUI cycles, we found that the intervention was associated with a higher CPR and ChPR (low inconsistency, I2 = 0%). In fresh-ET or frozen-ET groups, there was a higher rate of ChPR in Sildenafil co-treatment women.

A subgroup analysis for the evaluation of the way of Sildenafil administration was possible only for the IVF Fresh-ET/Frozen-ET groups. Even if only one study evaluated the oral administration of Sildenafil in these groups [42], vaginal administration seems to be more effective, but further studies are needed to confirm these results.

A subgroup analysis revealed that also timing of administration had a significant effect. In fact, only the delayed starting of Sildenafil administration during the TI and IUI cycles led to a thicker endometrium and to a higher biochemical pregnancy rate.

### 4.3. Interpretation

Based on our results, it would seem that ETh, CPR and ChPR are higher with the use of Sildenafil in women undergoing TI and IUI. In particular, a subgroup analysis evidenced that the delayed start of Sildenafil administration might significantly increase the chances of obtaining a thicker endometrium and pregnancy. Further studies are needed to reveal whether the way of Sildenafil administration has a significant effect on endometrial thickness and the pregnancy rate.

Considering women undergoing TI/IUI [19,37,39,40,41], in all except one trial [38], Sildenafil was orally administered. In this group, a significant improvement was highlighted for Eth, CPR and ChPR. Vaginal administration did not show significant advantages. This way of administration was evaluated in a single trial [38], but this trial compared Sildenafil to estrogen, while the other subgroup studies compared Sildenafil to no intervention.

Regarding the improvement of Eth in women undergoing MAR treated with Sildenafil, it is known that this molecule improves artery blood flow through the prevention of cGMP breakdown [18]. This leads to an increase in smooth muscle relaxation and vasodilation. The possible explanation of the impact of the Sildenafil on the endometrium is that this molecule exerts a positive effect on endometrial growth increasing endometrial vascularization through the described method. This mechanism acts in synergy with estrogens that secrete angiogenic factors to enhance revascularization [69,70]. This improvement in endometrial growth through an increased vascularization led to an improvement in the CPR and ChPR.

Better outcomes in terms of the CPR and ChPR were reported when Sildenafil supplementation was started 7–8 days from ovarian stimulation.

Physiologically, endometrial vascularization increases during the endometrial proliferative phase that generally last from the 7th day of the menstrual cycle under the influence of estrogens through the action of different angiogenic factors [69,70,71]. As natural killer cells release cytokines that are involved in embryo implantation failure through nitric oxide action, it may be beneficial to limit Sildenafil administration only when spiral arteries have already formed to avoid a high concentration of nitric oxide [72]. In fact, if the assumption of Sildenafil administration is to increase the endometrial vascularity, the delayed administration of this molecule adapts better to the physiology of the endometrial cycle, acting in concert with the increase in estrogen.

Considering women undergoing fresh or frozen-ET [12,42,43,44,45,46], vaginal Sildenafil represented the most common way of administration, and only one trial administered oral Sildenafil [42]. In this group, a significant improvement in ETh was not highlighted in patients treated with Sildenafil. Nevertheless, in treated women, a higher pregnancy rate was present. The only trail that considered oral administration in women undergoing the described ART techniques [42] showed a significant improvement in ETh but not in the pregnancy rate. However, considering women undergoing fresh- or frozen-ET, only the ChPR improvement was reported and not the CPR.

Regarding the non-improvement of Eth in women undergoing ART treated with Sildenafil, different explanations can be provided for patients undergoing fresh-ET and for patients undergoing frozen-ET. Women undergoing controlled ovarian stimulation achieve an increase in peak serum estradiol levels up to 10–12 times higher [73]. Since estradiol acts on the growth of the endometrium, the maximum effect on endometrial thickness can be obtained with only the high concentration of estrogen due to ovarian stimulation.

Instead, all women evaluated in the frozen-ET groups underwent an endometrial preparation with an artificial cycle. The dynamics of action of the estrogens administered in the artificial cycles of endometrial preparation on the endometrium are not fully known. It is possible that in this type of treatment, the synergy is present in MAR treatments in which Sildenafil acts in concert with estrogen to increase endometrial vascularization and therefore increase its thickness is lost.

Improvement in the ChPR was reported when the fresh-ET and frozen-ET groups were analyzed together. However, there was no significant improvement when the two groups were analyzed separately. The lack of improvement in the analysis of the frozen-ET group alone may be due to the number of limited studies available. In frozen-ET, the supplementation of Sildenafil might be useful to reach the maximum effect on endometrial vascularization, while maybe this is not possible in women undergoing fresh-ET who have higher levels of estrogens. Further studies are necessary to prove this hypothesis.

In summary, a biological explanation of the apparently different efficacies of Sildenafil among women undergoing TI and IUI compared to women undergoing IVF and fresh or frozen-ET might lie on the different levels of estrogen that are achieved during the different types of treatments [74,75]. In fact, as previously reported, estrogen treatment is an option for achieving a thicker endometrium, and high levels of endogenous estradiol might act as a cotreatment. This aspect may explain the non-significant results in endometrium thickness obtained in women undergoing transfer after treatments that required higher estradiol levels. Moreover, in our meta-analysis, the pregnancy rate resulted higher in Sildenafil treated women (*p* = 0.05), probably due to higher vascularization and, therefore, higher receptivity.

Regarding the route of administration, further studies are needed to evaluate the efficacy of the vaginal route of administration of sildenafil in women undergoing ART. This route of administration could ensure a higher endometrial concentration of sildenafil. In fact, vaginal absorption occurs through the vaginal mucosa which is highly vascularized, and it does not depend on food intake and avoids hepatic metabolism.

### 4.4. Strengths and Limitations

The present meta-analysis is the only one available on this issue. However, outcomes were calculated by pooling the results of a small number of studies and a small number of patients. Moreover, a certain heterogeneity across studies was present in terms of inclusion criteria (exclusion of uterine pathologies), Sildenafil therapy (way, dose and timing of administration) and population characteristics.

## 5. Conclusions

While the data from this study support a positive impact of sildenafil on ETh and pregnancy rate in women undergoing timed intercourses, Sildenafil therapy does not appear to improve outcomes in women undergoing IUI and IVF treatments. In fact, available data support a positive impact of Sildenafil on ETh and the pregnancy rate only in women undergoing timed intercourses, specifically when it is administered after day 7 of the cycle. Understanding endometrial angiogenesis mechanisms and the roles of angiogenic factors during ART treatments might provide new insights and clarify the effect of Sildenafil on the endometrium during different phases of the endometrial cycle. Due to the limitations of available studies, further RCTs are still mandatory in order finally to confirm or not the real clinical effectiveness as well as to establish the best timing, dose and duration of Sildenafil administration.

## Figures and Tables

**Table 1:** Data about general features of the manuscripts evaluated in this systematic review and meta-analysis including the dose and the days of Sildenafil treatment in intrauterine insemination cycles/timed intercourse cycles.

Author and Year	Study Design and Time of Realization	Participants and Main Inclusion Criteria (Number)	Enrollment Criteria	Controlled Ovarian Stimulation Protocols	Intervention Group	Main Finding
Kortam, M.A.M.F. et al., 2019	- Type: Single-center RCT - Duration: from October 2017 to May 2018 - Randomization: closed envelope method - Blinding: not reported - Number of protocol: NCT03301233	90 patients undergoing timed intercourse Women: - Total: 90 - Treatment 45 - Control 45	- unexplained infertility - Age 18–35 years - BMI < 30 - ovulatory cycles - No presence of any organic lesion of uterus, ovaries or tubes	- CC 100 mg/day starting from day 2 of the cycle for 5 days - oral E2 2 mg 2X/die from day 2 of the cycle till hCG - U-hCG (5000 UI) at leading follicle size of 18 mm - Luteal phase support with oral progesterone from day of ovulation (Dufaston 10 mg X2/daily)	Treatment group: - Treatment: Sildenafil oral, 75 mg/day - Timing: from cycle day 2 to HCG administration Control group: - Treatment: placebo - Timing: from cycle day 2 to HCG administration	Significant improvement of endometrial thickness and pattern in Sildenafil treated women No other significant beneficial effects
Mohamed, T.Y., 2019	- Type: Single-center RCT - Randomization: simple - Blinding: not reported	40 patients undergoing timed intercourse Women: - Total: 40 - Treatment 20 - Control 20	- anovulatory infertility - Age: 18-40-years - polycystic ovary - normal baseline FSH and LH - normal uterus - no prior ovarian or adnexal surgery or organic pelvic pathology	- letrozole 5 mg from 3rd to 7th day of the cycle - U-hCG (10,000 UI)	Treatment group: - Treatment: Sildenafil oral, 50 mg/day - Timing: from cycle day 8 to hCG administration Control group: no intervention	In Sildenafil treated group: - Significant improvement of endometrial thickness - Significantly higher clinical pregnancy rate
Fahmy, A.A. et al., 2015	- Type: Single-center RCT - Duration: from January to July 2012 - Randomization: sealed envelope - Blinding: not reported	70 patients undergoing timed intercourse Women: - Total: 70 - Treatment 35 - Control 35	- primary or secondary infertility - Age: 18-40 years - Regular menstrual cycles - No ovarian cysts, abnormal hormonal profile, history of any pelvic pathology	- clomiphene citrate 150 mg from 3rd to 7th day of the cycle - U-hCG (5000 UI)	Treatment group: - Treatment: Sildenafil oral, 75 mg/day - Timing: from cycle day 7 to day 11 of the cycle Control group: - Treatment: placebo - Timing: from cycle day 7 to day 11 of the cycle	In Sildenafil treated group: - Significant improvement of endometrial thickness - Significantly higher chemical pregnancy rate
Mangal, S., Mahirishi, S., 2016	- Type: Single-center RCT - Duration: from July 2014 to April 2015 - Randomization: not reported - Blinding: not reported	100 patients undergoing Intra Uterine Insemination Women: - Total: 100 - Treatment 50 - Control 50	- thin endometrium (Day 8 ET <7mm) - Age: <40 years	clomiphene citrate or gon-adotropin	Treatment group: - Treatment: Sildenafil vaginal, 100 mg/day - Timing: from cycle day 8 of the cycle Control group: - Treatment: E2 2 mg 6–8 hourly	Significantly higher cumulative clinical pregnancy rate (3 intrauterine insemination cycles). Not significant improvement of endometrial thickness
Vardhan, S. et al., 2019	- Type: Single-center RCT - Duration: 2 years - Randomization: not reported - Blinding not reported	80 women undergoing timed intercourse Women: - Total: 80 - Treatment 40 - Control 40	- Endometrial thickness of <7 mm on the day of ovulation - Age: 35 years - No organic pelvic pathology, congenital uterine anomaly, acquired deformities of uterus (Asherman syndrome)	clomiphene citrate 50 mg from 2nd to 6th day of the cycle estradiol valerate tablets orally by the step-up method: from the first day to the 4th day, 2 mg estradiol valerate tablets, and from the 5th to the 8th day, 4 mg estradiol tablets and from the 9th to the 12th day of the menstrual cycle, 6 mg estradiol valerate were given daily	Treatment group: - Treatment: Sildenafil oral, 25 mg/day - Timing: from cycle day 1 of the cycle to day 12 Control group: no intervention	In Sildenafil treated group: - Significant improvement of endometrial thickness - Significantly higher clinical pregnancy rate.
Reddy, L.P. et al., 2016	- Type: Single-center RCT - Duration: 4 months - Randomization: not reported - Blinding not reported	80 women undergoing timed intercourse Women: - Total: 80 - Treatment 40 - Control 40	- Age: <40 years - Regular menstrual cycles - No organic pelvic pathology - No endocrine disorders except thyroid disorder	clomiphene citrate 100 mg from 3rd to 7th day of the cycle	Treatment group: - Treatment: Sildenafil oral, 50 mg/day - Timing: from cycle day 8 of the cycle Control group: no intervention	In Sildenafil treated group: - Significantly improvement of endometrial thickness - Significantly higher chemical pregnancy rate.

**Table 2:** Data about general features of the manuscripts evaluated in this systematic review and meta-analysis including the dose and the days of Sildenafil treatment in IVF fresh and frozen embryo transfer cycles.

Author and Year	Study Design and Time of Realization	Participants and Main Inclusion Criteria (Number)	Enrollment Criteria	Controlled Ovarian Stimulation Protocols	Intervention Group	Main Finding
Moini, A. et al., 2020	- Type: Single-center RCT - Duration: February 2014 to November 2016 - Randomization: random allocation sequence generated by a randomized block design - Blinding: double-blind - Number of protocol: NCT03192709	66 patients undergoing IVF fresh cycles Intention to treat: - Total: 66 - Treatment A: 22 - Treatment B: 22 - Control: 22 Per protocol: - Total: 66 - Treatment A: 21 - Treatment B: 18 - Control: 17	- AMH >1.5, FSH < 10 - at least 2 consecutive failed IVF-ET cycles with at least a transfer of two good quality embryos - hCG day endometrial thickness <7 mm in all prior IVF/ICSI attempts - Age ≤ 38 years - No history of myomectomy or Asherman’s syndrome - normal endometrial appearance	- GnRH-ag long protocol - FSH/FSH +LH - U-hCG (10000 IU) at follicle size 18mm (≥2). - Oocyte retrieval 36 h after hCG - 2/3 days embryos transfer - Luteal phase support with progesterone (100 mg IM daily or 800 mg vaginal daily)	Treatment group A: - Treatment: Sildenafil vaginal, 100 mg/day - Timing: from cycle day 1 to OPU Treatment group B: - Treatment: Placebo then Sildenafil vaginal, 100 mg/day - Timing: placebo from cycle day 1 to 2 days before hCG administration, then Sildenafil to to OPU Control group: - Treatment: Placebo - Timing: from cycle day 1 to OPU	No significant beneficial effects
Shahrokh, T.N.E. et al., 2018	- Type: Single-center RCT - Randomization: simple - Blinding: not reported	72 patients undergoing IVF fresh cycles Women: - Total: 72 - Treatment 36 - Control 36	- ≥2 failed IVF- ET cycles - Age <45 years (21–43)	- GnRH-ag long protocol Buserelin 1.5 mg daily - FSH (dose according to patients’ characteristics)	Treatment group: - Treatment: Sildenafil vaginal, 100 mg/day - Timing: from cycle day 2 to HCG administration Control group: no intervention	Significant improvement of endometrial thickness in Sildenafil treated women No other significant beneficial effects
Ataalla, W.M. et al., 2016	- Type: Single-center RCT - Duration: from January 2012 to January 2014 - Randomization: women asked to choose a number from 1 to 60 - Blinding: double-blinded	60 patients undergoing IVF fresh cycles Women: - Total: 60 - Treatment 30 - Control 30	- Age ≤ 35 - No ovarian surgery - Low responders to COH: ≤ 3 follicles on the day of hCG administration or ≤ 3 oocytes - previous cycle cancellation due to poor follicular development	- GnRH-antagonist protocol - FSH 300 IU + 150 hMG from 2nd day of cycle, dose adjusted according patient characteristics - U-hCG (10,000 UI) at leading follicle(s) size of 17 mm - Oocyte retrieval 34–36 h after hCG - Day 3 embryos transferred - Luteal phase support with progesterone (150 mg IM daily)	Treatment group: - Treatment: Sildenafil oral, 50 mg/day - Timing: from cycle day 1 Control group: - Treatment: Placebo oral - Timing: from cycle day 1	Significant improvement of endometrial thickness in Sildenafil treated women. No other significant beneficial effects.
Firouzabadi, R.D. et al. 2013	- Type: Single-center RCT - Duration: from 2009 to 2011 - Randomization: random numbers tables - Blinding: not reported - Number of protocol: IRCT201210232575N3 NCT01668446	80 patients undergoing FET cycles Women: - Total: 80 - Treatment 40 - Control 40	- an antecedent of poor endometrial response - high-grade embryos - Age < 40 years - No history of endocrine diseases -No hysteroscopic surgeries.	- estradiol by a step-up method while in menstruation - 3 days high-grade embryos transferred - Luteal phase support with progesterone (100 mg IM daily) - E2 and P4 were measured in an hour after first P4 injection	Treatment group: - Treatment: Sildenafil vaginal, 50 mg/day - Timing: during endometrial preparation until the start P4 administration Control group: no intervention	In Sildenafil treated group: - Significant improvement of endometrial thickness and pattern - Significantly higher chemical pregnancy rate
Check, J.H. et al., 2004	Type: Single-center RCT - Randomization: random numbers table - Blinding: not reported	20 patients undergoing FET cycles Intention to treat: - Total: 20 - Treatment 10 - Control 10 Per protocol: - Total: 16 - Treatment 9 - Control 7	- failed to attain an 8 mm endometrial thickness with oral E2	- E2 oral regimen Step up method: E2 2 mg × 5 days, 4 mg × 4 days, 6 mg × 5 days - Luteal phase support with progesterone: 200 mg twice daily vaginal suppositories and 100 mg in oil daily	Treatment group: - Treatment: Sildenafil vaginal, 100 mg/day - Timing: from day 3 to day 9 of endometrial preparation Control group: Vaginal E2 2 mg 2× per day Timing: from day 2 to peak thickness	No significant beneficial effects
Kahsouh, A.M. et al., 2017	Type: Single-center RCT - Duration: Jun 2015-Dec. 2016 - Randomization: simple - Blinding: not reported	90 patients undergoing FET cycles Women: - Total: 90 - Treatment 45 - Control 45	- an antecedent of poor endometrial response and frozen embryos 2 mg of estradiol valerate 6–8 hourly from the day 2–14 of the menstrual cycle	- estradiol valerate 2 mg, every 6–8 h from the day 2 to day 14 of the menstrual cycle - Luteal phase support with progesterone: 400 mg pessaries vaginal 3 days prior embryo transfer	Treatment group: - Treatment: Sildenafil vaginal, 100 mg/day - Timing: from day 2 of menstrual cycle and discontinued 48–72 hours prior to the embryo transfer. Control group: E2: 2 mg every 6–8 h	Significant improvement of endometrial thickness and patternNo other significant beneficial effects

## Data Availability

The datasets used and/or analyzed during the current study are available from the corresponding author on reasonable request.

## Supplementary Materials

The following are available online at https://www.mdpi.com/article/10.3390/jcm10194346/s1, Figure S1: a Forest plot of comparison, subgroup analysis according to time of administration: endometrial thickness (ETh) according to group allocation—intervention (Sildenafil) versus control in insemination and timed intercourse cycles. b Forest plot of comparison, subgroup analysis according to time of administration: chemical pregnancy rate (ChPR) according to group allocation—intervention (Sildenafil) versus control in intrauterine insemination and timed intercourse cycles. Figure S2: a Forest plot of comparison, subgroup analysis according to administration route: endometrial thickness (ETh) according to group allocation—intervention (Sildenafil) versus control in IVF fresh and frozen embryo transfer. b Forest plot of comparison, subgroup analysis according to administration route: chemical pregnancy rate (ChPR) according to group allocation—intervention (Sildenafil) versus control in IVF fresh and frozen embryo transfer.
