# Supplementary figures and images for: Sildenafil Supplementation for Women Undergoing Infertility Treatments: A Systematic Review and Meta-Analysis of Randomized Controlled Trials
Source: J Clin Med. 2021 Sep 24;10(19):4346. doi: 10.3390/jcm10194346 (PMC8509188; doi:10.3390/jcm10194346)

a. Endometrial Thickness

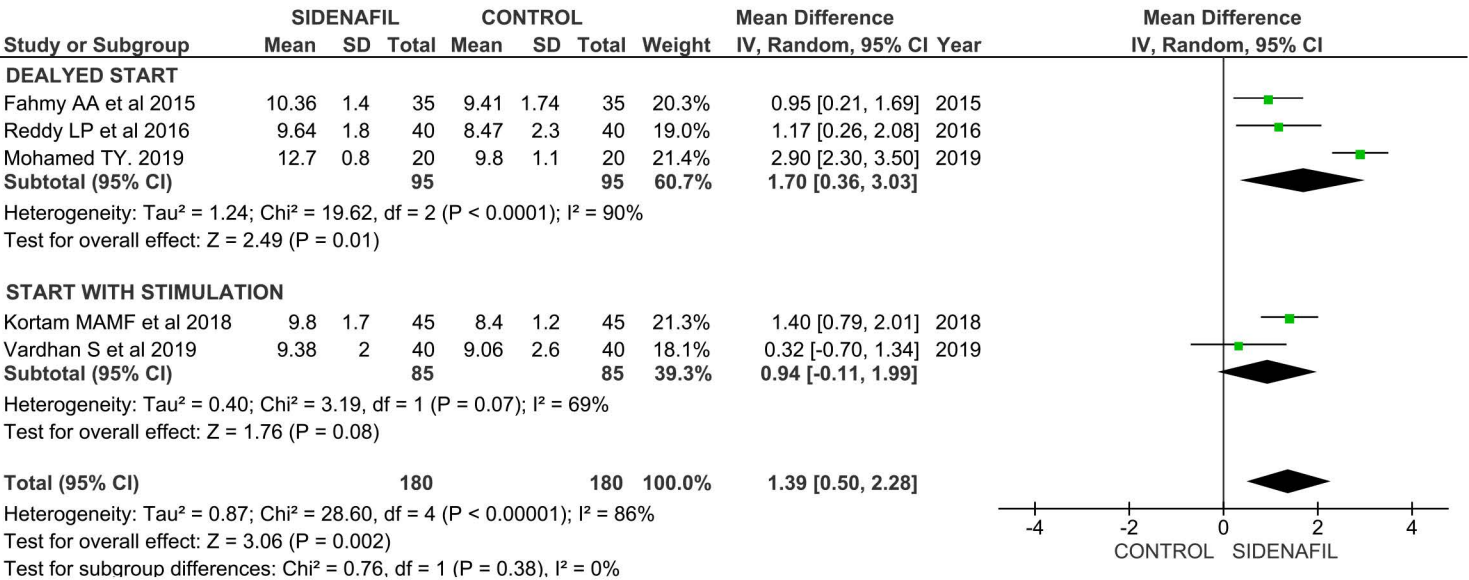

b. Chemical Pregnancy Rate

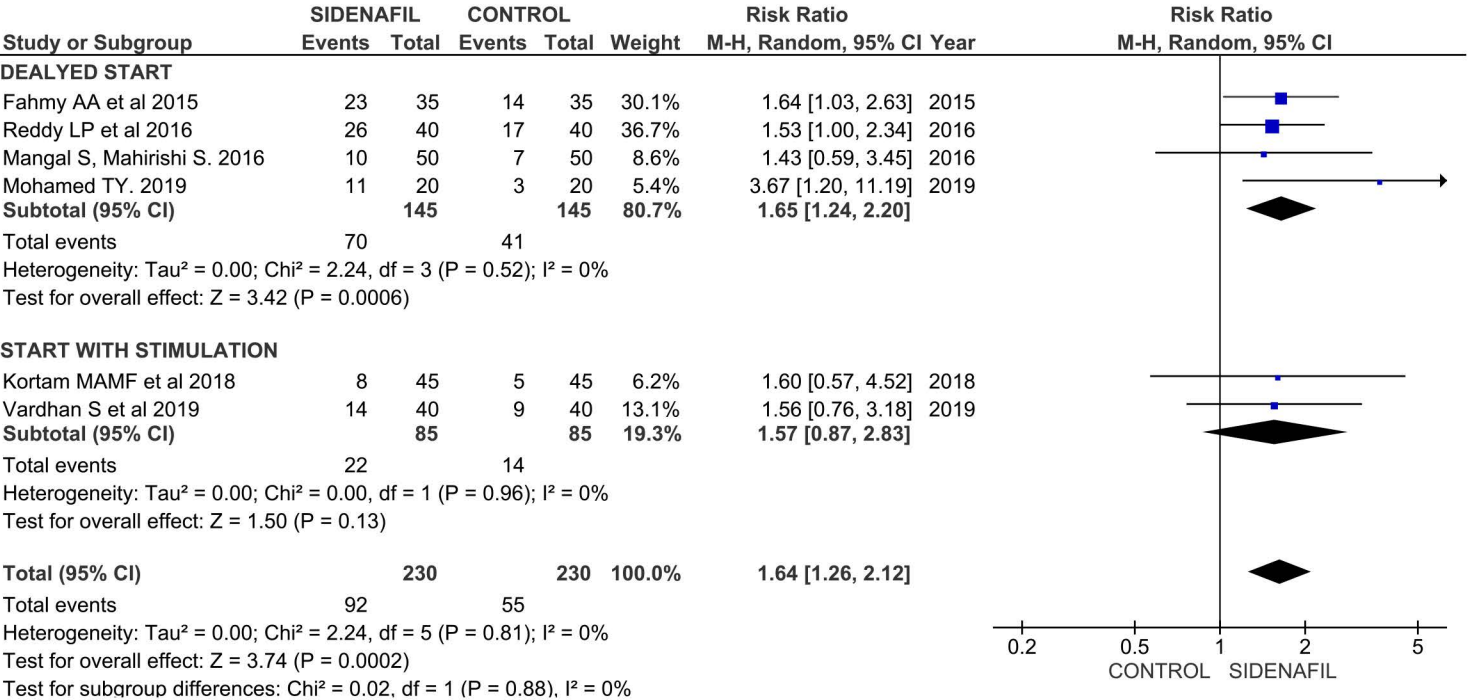

Supplement: Supplementary file 1 [file jcm-10-04346-s001.zip › Supp FIGURE S1-rev.pdf]

a. Endometrial Thickness

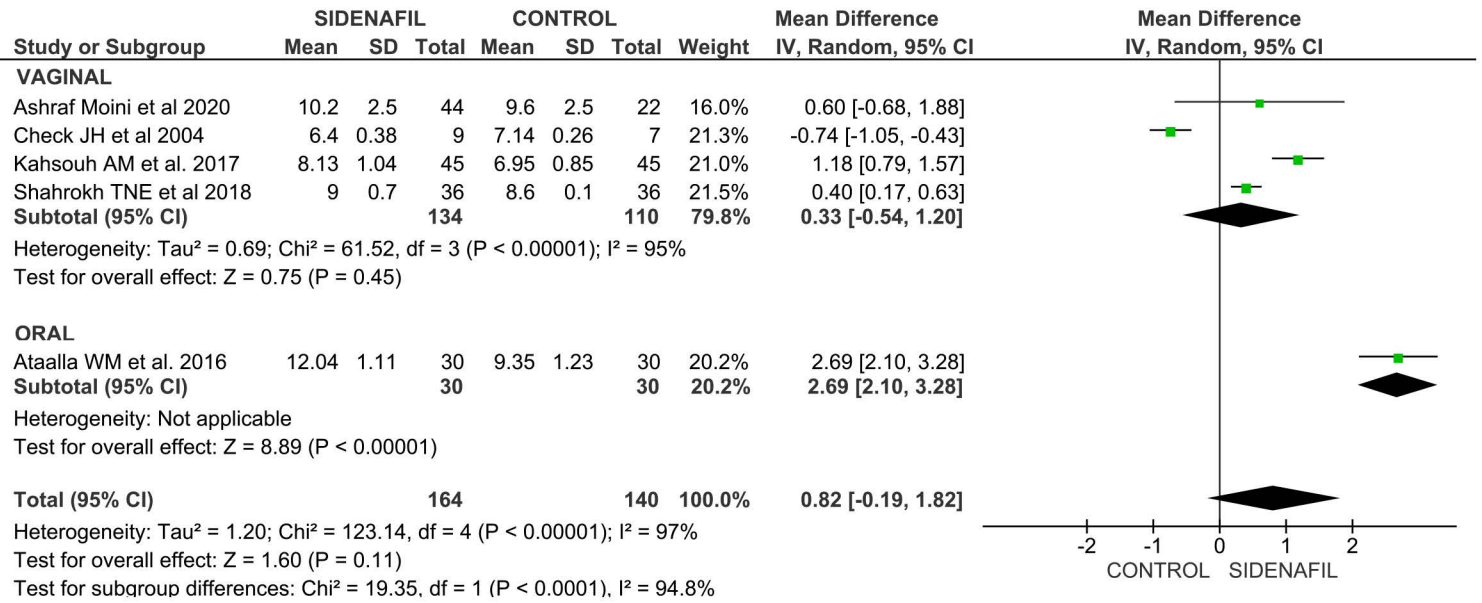

b. Chemical Pregnancy Rate

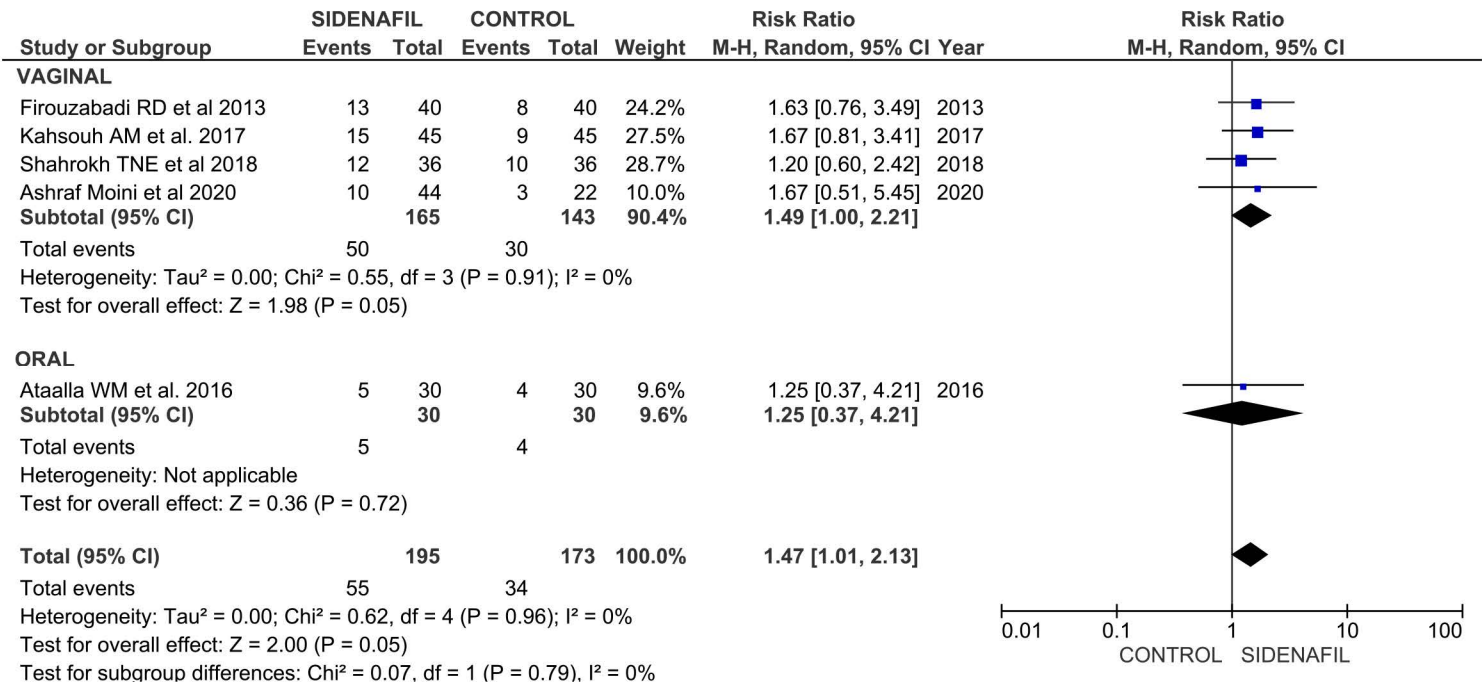

Supplement: Supplementary file 1 [file jcm-10-04346-s001.zip › Supp FIGURE S2-rev.pdf]
